# High SRC-1 and Twist1 expression predicts poor prognosis and promotes migration and invasion by inducing epithelial-mesenchymal transition in human nasopharyngeal carcinoma

**DOI:** 10.1371/journal.pone.0215299

**Published:** 2019-04-11

**Authors:** Jingchun Zhou, Jingjing Zhang, Ming Xu, Zhaoyang Ke, Wei Zhang, Jiahao Mai

**Affiliations:** 1 Department of Otorhinolaryngology, The Second Clinical Medical College of Jinan University, Shenzhen People's Hospital, Shenzhen, China; 2 Department of Otorhinolaryngology, Peking University Shenzhen Hospital, Shenzhen, China; 3 Department of Otorhinolaryngology, The Affiliated Hospital of Medical School, Ningbo University, Zhejiang, China; Seoul National University College of Pharmacy, REPUBLIC OF KOREA

## Abstract

Steroid receptor coactivator 1 (Src-1) and Twist1 are aberrantly upregulated in a variety of tumors and play an important role in tumor progression. However, the exact role of Src-1 and Twist1 in nasopharyngeal carcinoma (NPC) is uncertain. In this study, we investigated the possible prognostic value and biological effect of Src-1 and Twist1 in NPC. Src-1 and Twist1 expression was detected in a cohort of NPC patients (n = 134) by qRT-PCR. Kaplan-Meier survival analysis was used comparing overall survival (OS) and progression-free survival (PFS). Multivariate analysis was performed using the Cox proportional hazard regression model. Biologic effect of Src-1 and Twist1 in NPC cell lines was evaluated by western blot, colony formation assay, soft agar assay, scratch wound healing assay, transwell invasion assay and tumor xenografts growth. We have found that Src-1 and Twist1 were aberrantly upregulated in human NPC tissues, and associated with advanced tumor stage, distant metastasis and unfavorable prognosis. Knockdown of Src-1 or Twist1 in human NPC cell line CNE-1 suppressed colony formation, anchorage-independent growth, cell migration, invasion and tumor xenografts growth, while enforced expression of Src-1 or Twist1 in human NPC cell line HNE-2 promotes anchorage-independent growth, cell migration and invasion. In addition, Src-1 and Twist1 could suppress E-cadherin expression and increase Vimentin expression, thus suggested that Src-1 and Twist1 enhanced the malignant behaviors of NPC cells via inducing epithelial-mesenchymal transition (EMT). Our data indicated that Src-1 and Twist1 could be possible prognostic biomarkers and potential therapy targets for patients with NPC.

## Introduction

Nasopharyngeal carcinoma (NPC), a unique malignancy arising from the epithelium of nasopharynx, is characterized by its unique geographic distribution [1]. NPC has the highest incidence in southern China, Southeast Asia and North Africa, but it is rare in the rest part of the world [2]. According to global cancer statistics from the International Agency for Research on Cancer, nearly 80% new NPC cases in 2008 were in Asia and only 5% were in Europe. Several factors have been implicated in the development of NPC, including genetic susceptibility, Epstein-Barr virus (EBV) infection and chemical carcinogens [3–5]. Besides, NPC is a poorly or undifferentiated carcinoma. It has high radio- and chemosensitivity, and a great propensity for distant metastasis [6]. Thus, radiotherapy is recommended for the treatment of nonmetastatic disease and has a high cure rate for patients with low NPC stages. However, the majority of NPC patients are diagnosed with locally advanced stages. Various modes of combined chemoradiotherapy have been used to treat these NPC patients, but the 5-year overall survival rate were only 53%-80% and 28%-61% in NPC stages III and IV, respectively [7, 8]. To date, genomic abnormalities of NPC remain obscure and no targeted therapy has been established. Therefore, it is urgent to find molecular targets which can predict prognosis or guide for targeted therapy in NPC.

The first nuclear receptor coactivator, steroid receptor coactivator 1 (SRC-1, also known as NCOA1) was identified in a yeast two-hybrid system in 1995 as an enhancer of the progesterone receptor [9]. Virtually all transcription factors in mammals execute their transcriptional activation functions with coactivators. In human, SRC-1 was proved to strongly potentiate the transcriptional activities of progesterone receptor, estrogen receptor α and many other nuclear receptors in a ligand-dependent manner. In addition, SRC-1 has shown to interact with other transcriptional factors such as AP-1, Ets2, NFκβ, PEA3 and HOXC11 [10–12]. Through modulating gene expression regulated by nuclear receptors or other transcriptional factors, SRC-1 plays a crucial role in cell proliferation, differentiation, carcinogenesis and metastasis [13, 14]. Moreover, insights from clinical data suggested that Src-1 was significantly upregulated in many cancers, such as breast cancer [15, 16], prostate cancer [17] and thyroid cancer [18].

Twist1, a basic helix-loop-helix (bHLH) domain containing transcription factor, was originally identified in Drosophila as an essential regulator in embryogenesis [19]. During embryo development, Twist1 is essential for mesoderm formation, specification and differentiation. Moreover, Twist1 is found to overexpress in a variety of tumors and plays an important role in cancer initiation, progression and metastasis [20]. Previous studies have shown that increased Twist1 expression is associated with breast cancer invasion and metastasis [21]. Epithelial-mesenchymal transition (EMT) is crucial for cancer progression and characterized by downregulation of E-cadherin and upregulation of N-cadherin and Vimentin. In hepatocellular carcinoma, Twist1 suppresses E-cadherin expression and induces EMT changes [22]. Down-regulation of Twist1 in androgen independent prostate cancer cells increased anticancer drug sensitivity and suppressed cell migration and invasion [23]. Twist1 overexpression in gastric cancer cell line BGC-823 increased cell migration and decreased drug sensitivity to arsenic oxide [24]. Moreover, Twist1 was shown to suppress oncogene-induced and p53-dependent cellular senescence [25].

Twist1 is upregulated by a variety of factors in cancer, including SRC-1, STAT3, HIF-1α and NF-κB. Among them, SRC-1 serves as a coactivator for transcription factor PEA3 to enhance Twist1 expression, indicating that SRC-1 promotes breast cancer invasiveness and metastasis by upregulating Twist1 expression [26]. Furthermore, SRC-1 and Twist1 expression in breast cancer was positively correlated with a poor prognosis [27]. In this study, we investigated the expression of SRC-1 and Twist1 in human NPC patients and its correlation with clinicopathological parameters. We found that high expression of Src-1 and Twist1 was associated with poor prognosis. NPC cell line CNE-1 and HNE-2 were used to study the influence of Src-1 and Twist1 on colony formation, anchorage-independent growth, cell migration and invasion. We found that Src-1 and Twist1 played an important role in anchorage-independent growth, cell migration and invasion. Our findings suggested that Src-1 and Twist1 could be promising prognostic markers for NPC patients.

## Materials and methods

### Patient and tissue samples

Written informed consent was obtained from all participants in our study. The use and collection of tissue samples were reviewed and approved by the ethics committee of the Shenzhen People's Hospital. A retrospective analysis of one hundred and thirty-four newly diagnosed, non-metastatic NPC patients from January 2010 to July 2013 were enrolled in this study. All patients underwent surgery treatment followed by adjuvant radiotherapy and/or chemotherapy, and the tumor samples and paired normal tissues were collected. The clinical stage was determined according to the 7^th^ edition UICC/AJCC cancer staging system. Clinical data were collected from the hospital database and the follow-up for survival analysis were conducted for up to 48 months after surgery.

### Cell culture

NPC cell line S18, HONE-1, C666-1, CNE-1 and HNE-2 were obtained from the Peking University Shenzhen Hospital. HEK-293T was purchased from the American Type Culture Collection (ATCC, Rockville, MD). S18, HONE-1, C666-1, CNE-1, HNE-2 and HEK-293T were cultured in Dulbecco's Modified Eagle Medium (DMEM) (Invitrogen, USA) supplemented with 10% fetal bovine serum (HyClone, USA) and 1% PenStrep (100 U/mL Penicilium and 100 μg/mL Streptomycin) in a humidified 5% CO2 atmosphere at 37°C.

### Quantitative real-time polymerase chain reaction (qRT-PCR)

Total RNA of tissue samples was extracted according to the operation protocol of TRIzol reagents (Invitrogen, California, USA). The quality and quantity of extracted RNA was evaluated by Nanodrop 2000 spectrophotometer. Complementary DNA was generated using RevertAid First Strand cDNA Synthesis kit (Thermo, Massachusetts, USA) and quantified using a standard SYBR-Green PCR kit protocol (Takara, Japan) in ABI 7900 Real Time PCR system. All samples were done in triplicate and normalized to GAPDH. The relative expression levels were calculated by the equation 2^−ΔΔCT^. The primers for qRT-PCR were: Src-1: Forward: 3’-TCA CTT CAG TCC GCC ACT-5’; Reverse: 3’-TCG CCT GTT CCT GGT TGT-5’; Twist1: Forward: 3’-ACC ATC CTC ACA CCT CTG-5’; Reverse: 3’-GAT TGG CAC GAC CTC TTG-5’. The log2-transformed mRNA expression fold change of Src-1 and Twist1 when comparing tumor versus normal tissues was calculated by equation: Log2 (T/N) = log2 (Src-1 or Twist1 mRNA expression in tumor/ Src-1 or Twist1 mRNA expression in paired normal tissue).

### Immunohistochemistry (IHC)

The formalin-fixed paraffin-embedded tissue samples were cut into 4 μm thick section. Then sections were deparaffinized in xylene and rehydrated through graded alcohol. After antigen retrieval in citrate buffer, the sections were incubated with 3% H_2_O_2_ for 30 min followed by 5hr blocking in 20% goat serum in PBS. Then the sections were incubated with SRC-1 (128E7) Rabbit mAb (Cell signaling #2191; 1: 200) or Anti-Twist antibody (Abcam #ab50581; 1: 500) at 4°C overnight. Species-appropriate biotinylated secondary antibodies were used for antigen detection. Human Anti-rabbit IgG (H+L) (Cell signaling#14708) was used as negative control to exclude unspecific staining. The score of IHC staining was evaluated by two experienced pathologists independently. Staining intensity was divided into four grades: 0 (negative), 1 (weakly positive), 2 (moderately positive), and 3 (strongly positive). The percentage of positive cells was classified into five grades: 0 for 0–5%, 1 for 6–25%, 2 for 26–50%, 3 for 51–75%, and 4 for > 75%. The final score was obtained by multiplying the staining intensity score and grades for percentage of positive cells. A final score of 0–3 was considered no expression, 4–6 was low expression, 7–9 was middle expression and 10–12 was high expression.

### Plasmid constructs, lentivirus packaging and infection

Src-1 and Twist1 expression plasmids were constructed by inserting the coding sequence of Src-1 or Twist1 into the pCDH-CMV-MCS-EF1-Puro (System Biosciences #CD510B-1) lentiviral vector. Short hairpin RNA (shRNA) constructs for Src-1 and Twist1 (sh-Src-1 and sh-Twist1) were purchased from Sigma-Aldrich (Carlsbad, CA, USA). HEK-293T cells were seeded at 3×10^6^ cells in 10 cm culture dishes for 48h, then transfected with Src-1, Twist1, sh-Src-1 and sh-Twist1 expression plasmids plus lentivirus packaging vectors δ8.9 and VSVG using Lipofectamine 3000 (Life Technologies# L3000015) as protocols. The medium with virus particles were collected at 24 h, 48 h and 72 h after transfection and stored at -80°C. To knockdown or overexpress Src-1 and Twist1, CNE-1 and HNE-2 were seed at 2.5×10^5^ cells per well in 6-well-plates for 24h, then incubated with 1ml medium with virus particles overnight plus with 8ug/ml polybrene (Sigma-Aldrich, Carlsbad, CA, USA). Successfully infected cells were selected by 1 ug/ml puromycin for 4 days post infection.

### Colony formation assay and soft agar assay

To evaluate the colony formation ability, CNE-1 cells transfected with sh-Src-1, sh-Twist1 or sh-NC control were seed at 500 cells per well in 6-well-plates and cultured for 2 weeks. Then cells were fixed by 4% PFA for 15 min and stained with crystal violet staining solution (Beyotime #C0121). Soft agar assay was done as previous reports [28]. Briefly, CNE-1 and HNE-2 were seeded in 0.4% top agar at 5000 cells per well in 6-well-plates. Then supplemented with 250 μl culture medium twice weekly and cultured for 3 weeks. Colonies were stained by incubating with 1mg/ml thiazolyl blue tetrazolium bromide (MTT) solution for 3hr.

### Cell migration and invasion assay

Cell migration ability was evaluated by wound healing assay. CNE-1 and HNE-2 cells were seeded in 6-well-plates and when cell confluence reached 80%, the cell monolayer was scratched with a sterile plastic tip right in the middle. Then the cell debris was washed away with culture medium. Take photos of the plates at different time points under a microscope (IX71, Olympus, Tokyo, Japan). Digimizer software system (MedCalc software, Ostend, Belgium) was used to measure the distance between two edges of the scratch. Transwell cell invasion assay was performed using the 8 μm matrigel invasion chamber (BD Bioscience, San Jose, CA, USA) in 24-well plates. Briefly, 1×10^5^ cells were plated in upper chamber without serum and the lower chamber were filled with DMEM with 10% FBS. The invading cells were stained with Giemsa dye and imaged after incubating for 24 hr.

### Cell viability assay

Cell viability was determined according to the operation manual of CellTiter-Glo Luminescent Cell Viability Assay kit (Promega#G7572). CNE-1 cells transduced with sh-Src-1, sh-Twist1 or sh-NC control were seeded in 96-well-plate at 5000 cells per well. The cell viability was first tested 24 hr after seeding the cells as day 0 and tested every two days for 6d. All experiments were done in three duplicates.

### Western blot

Cells were lysed in RIPA buffer containing protease inhibitors (Sigma-Aldrich, Carlsbad, CA, USA). BCA protein assay kit (Thermo Scientific, Grand Island, NY, USA) was used to quantify protein concentration. A total of 30 ug protein was electrophoresed by 10% SDS-PAGE and transferred onto nitrocellulose membranes, then incubated with specific first antibodies overnight and corresponding second antibodies for 1 hr. The specific first antibodies were list as follows: SRC-1 (128E7) Rabbit mAb (Cell signaling #2191; 1: 1000); Anti-Twist antibody (Abcam #ab50581; 1: 2000), Vimentin (D21H3) XP Rabbit mAb (Cell signaling #5741; 1: 1000); E-Cadherin (24E10) Rabbit mAb (Cell signaling #3195; 1: 1000). The second antibody was goat anti-rabbit IgG HRP-linked antibody (Cell Signaling #7074; 1: 4000).

### Tumor Xenografts model

All animal experiments were approved by Animal Care and Experimental Committee of Shenzhen People's Hospital. BABL/c nude mice were housed in individually ventilated cages under specific pathogen free conditions. Mice were allowed access to sterilized water and feed *ad libitum*. The animal was monitored twice a week and their body weight were weighed. CNE-1 (2× 10^6^ cells) transduced with sh-Src-1, sh-Twist1 or sh-NC were subcutaneous injected into six-week old nude mice. Tumor volume were monitored every two days by calipers to calculate tumor volumes according to the formula: Tumor volume = (length × width^2^)/2. All mice were anaesthetized by inhalation of 3% isoflourane and decapitated four weeks after tumor cells injection. Then tumors were dissected out and weighed.

### Statistical analysis

Statistical analysis was performed by SPSS 19.0 software (IBM Corp, Armonk, New York, USA). The difference between two groups was analyzed by paired sample t-test. The difference of multiple groups was analyzed by one-way analysis. The correlation between Src-1 and Twist1 expression and clinicopathologic features was analyzed using chi-square test. Spearman analysis was used to evaluate the association between Src-1 and Twist1 expression. Survival curves were plotted using the Kaplan–Meier method and the log-rank test was used to compare the survival rates between groups with varying Src-1 and Twist1 expression levels. Multivariate analysis was performed by using the Cox proportional hazards model. All data was shown as mean ± standard deviation (x ± s). P<0.05 was considered statistically significant.

## Results

### Src-1 and Twist1 is aberrantly up-regulated in nasopharyngeal carcinoma tissues

**Fig 1:**
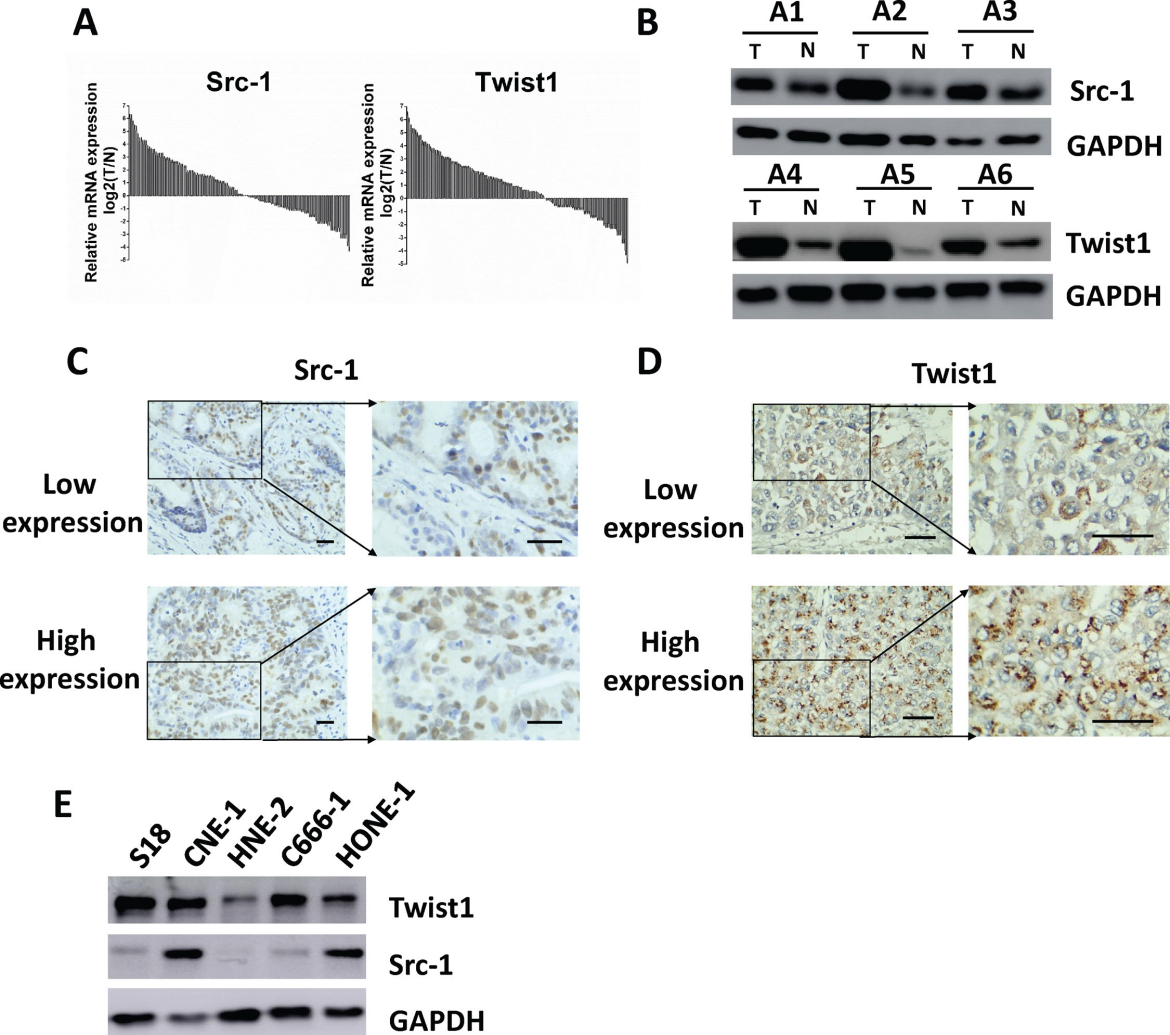
Src-1 and Twist1 is aberrantly up-regulated in nasopharyngeal carcinoma tissues. **(A)** the mRNA expression of Src-1 and Twist1 in tumor samples and adjacent normal tissues of the 134 NPC patients was evaluated by qRT-PCR, then relative mRNA expression Log2 (T/N) was calculated. **(B)** Src-1 or Twist1 expression in tumor samples (T) and paired normal tissues (N) of selected NPC patients was evaluated by western blot. **(C, D)** Representative images of IHC staining and statistical results for low and high expression of Src-1 or Twist1 in tumor tissues of NPC patients. Scale bars = 50μm. **(E)** Protein expression of Src-1 and Twist-1 in a panel of nasopharyngeal carcinoma cell lines.

The mRNA expression of Src-1 and Twist1 in tumor samples and adjacent normal tissues of the 134 NPC patients was evaluated by qRT-PCR. Log2-transformed mRNA expression fold change of Src-1 and Twist1 when comparing tumor versus normal tissues was shown in Fig 1A. The mean mRNA expression log2 (T/N) ratio of Src-1 and Twist-1 was 0.58 and 0.94. Src-1 and Twist1 were obviously up-regulated in 47.0% (63/134) and 48.5% (65/134) patients, respectively. Up-regulation of Src-1 or Twist1 was further verified by western blot (Fig 1B). The protein expression of Src-1 or Twist1 in tumor tissues of NPC patients was apparently overexpressed comparing with paired adjacent normal tissues. Additionally, IHC staining of Src-1 or Twist1 in tumor tissues demonstrated that Src-1 and Twist1 was highly expressed in NPC patients, too (Fig 1C and 1D). We also checked the protein expression of Src-1 and Twist1 expression in several NPC cell lines, and found that Src-1 and Twist1 were high expressed in CNE-1 and low expressed in HNE-2 (Fig 1E). In conclusion, Src-1 and Twist1 is aberrantly up-regulated in nasopharyngeal carcinoma tissues.

### High expression of Src-1 and Twist1 is associated with tumor stage and distant metastasis in nasopharyngeal carcinoma patients

The demographic and pathological characteristics of all subjects were list in Table 1. In this study, 75 males and 59 females were enrolled with a median age of 52.3±14.8 years (range from 35~73 years). All NPC patients were diagnosed with non-keratinizing carcinoma. There were 62 (46.3%) patients with stage I~II disease, and 72 (53.7%) patients with stage III~IV disease. EBV infection was found in 84 (62.7%) patients, and local disease recurrence was found in 32 (23.9%) patients. Lymph node metastasis and distant metastasis was found in 49 (36.6%) and 37 (27.6%) patients, respectively. To investigate whether high Src-1 or Twist1 expression is associated with any clinical feature, the 134 NPC patients was divided into high Src-1 group, low Src-1 group, high Twist1 group or low Twist1 group according to mRNA expression log2 (T/N) ratio of Src-1 and Twist1. The association between Src-1 or Twist1 expression with various clinicopathological parameters is shown in Table 1. High Src-1 expression is significantly associated with tumor stage (*P* = 0.038) and distant metastasis (*P* = 0.012). High Twist1 expression is closely correlated with tumor stage (*P* = 0.016), recurrence (*P* = 0.042) and distant metastasis (*P* = 0.001). Previous studies have demonstrated that SRC-1 serves as a coactivator for the transcription factor PEA3 to enhance Twist1 expression [26], thus we supposed a correlation between Src-1 and Twist1 expression in NPC patients. As we expect, Src-1 expression was closely associated with Twist1 (*P*<0.001) by spearman analysis, the correlation coefficient was 0.73 (Table 2).

**Table 1:** Associations between SRC-1 and Twist1 expression with clinicopathological characteristics in NPC patients.

Clinical parameters	No. of Patients	Src-1 expression			Twist1 expression		
		High	Low	*P* value	High	Low	*P* value
**Age**							
** >60**	41	21	20	0.575	16	25	0.189
** ≤60**	93	42	51		49	44	
**Sex**							
** Male**	75	31	44	0.164	36	39	1.000
** Female**	59	32	27		29	30	
**Histological grade**							
** Differentiated NKC**	28	15	13	0.524	12	16	0.631
** Undifferentiated NKC**	106	48	58		53	53	
**UICC/AJCC Stage**							
** I~II**	62	23	39	**0.038**	23	39	**0.016**
** III~IV**	72	40	32		42	30	
**EBV infection**							
** (+)**	84	40	44	1.000	46	38	0.075
** (-)**	50	23	27		19	31	
**Local recurrence**							
** Yes**	32	14	18	0.691	21	11	**0.042**
** No**	102	49	53		44	58	
**Lymph node metastasis**							
** Yes**	49	24	25	0.858	29	20	0.074
** No**	85	39	46		36	49	
**Distant metastasis**							
** Yes**	37	24	13	**0.012**	27	10	**0.001**
** No**	97	39	58		38	59	

NKC, non-keratinizing carcinoma.

**Table 2:** Correlation of SRC-1 and Twist1 expression in NPC by Spearman analysis.

		Src-1			Correlation coefficient	P value
		High expression	High expression	Total		
**Twist1**	**High expression**	44 (32.8%)	21 (15.7%)	65 (48.5%)	0.73	<0.001
	**Low expression**	19 (14.2%)	50 (37.3%)	69 (51.5%)		
	**Total**	63 (47.0%)	71 (53.0%)	134 (100.0%)		

### High expression of Src-1 and Twist1 is associated with unfavorable prognosis in nasopharyngeal carcinoma patients

**Fig 2:**
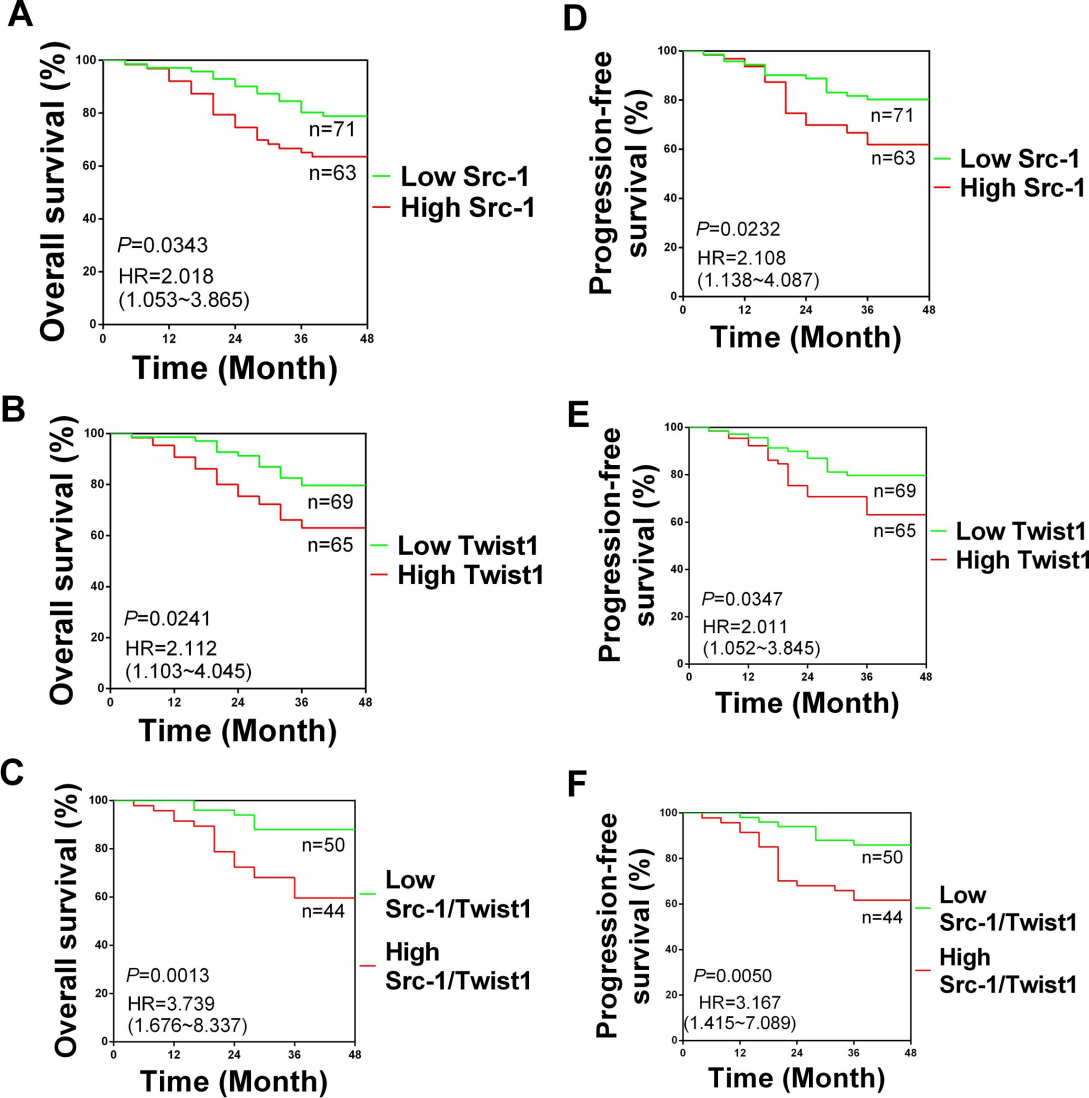
Kaplan-Meier survival analysis of NPC patients according to Src-1 and Twist1 expression. Overall survival according to Src-1 expression **(A)**, Twist1 expression **(B)** and both Src-1/Twist1 expression **(C)**. Progression-free survival according to Src-1 expression **(D)**, Twist1 expression **(E)** and both Src-1/Twist1 expression **(F)**.

The mean follow-up time was 40.9 months post-surgery (range from 4–48 months). The association between Src-1 and Twist1 expression with overall survival (OS) and progression-free survival (PFS) was examined. The OS and PFS rates in high Src-1 expression group (n = 63) were 61.9% and 67.6%, which were significantly lower than those of 78.9% and 80.3% in low Src-1 expression group (n = 71), respectively. The OS and PFS rates in high Twist1 expression group (n = 65) were 60.0% and 63.0%, which were apparently lower than those of 81.5% and 79.7% in low Twist1 expression group (n = 69), respectively. Analysis of Kaplan-Meier survival curves demonstrated that OS time of NPC patients from high Src-1 group and high Twist1 group was markedly worse than those from low Src-1 group and low Twist1 group (Fig 2A and 2D). Besides, PFS time of NPC patients from high Src-1 group and high Twist1 group was much worse than low Src-1 group and low Twist1 group, too (Fig 2B and 2E). These effects was significantly enhanced when comparing NPC patients with high Src-1/Twist1 expression and low Src-1/Twist1 expression (Fig 2C and 2F). Multivariate analysis identified old age, high Src-1 expression, high Twist1 expression and distant metastasis as independent predictive factors for the unfavorable OS time (Table 3). Furthermore, high Twist1 expression were independent predictive factors for the unfavorable PFS time (Table 3). These results suggested that Src-1 and Twist1 was potential biomarkers to predict unfavorable prognosis in NPC patients.

**Table 3:** Multivariate Cox proportional hazards model of predictive factors in OS and DFS.

Covariate	OS		DFS	
	HR (95% CI)	*P* value	HR (95% CI)	*P* value
**Age**				
** ≤60**	Reference		Reference	
** >60**	1.982 (1.133~3.645)	**0.021**	1.344 (0.860~2.565)	0.385
**Gender**				
** Female**	Reference		Reference	
** Male**	1.324 (0.654~2.325)	0.067	1.930 (0.922~3.856)	0.194
**Stage**				
** I~II**	Reference		Reference	
** III~IV**	1.765 (0.845~3.944)	0.188	1.655 (0.695~3.899)	0.305
**EBV infection**				
** (-)**	Reference		Reference	
** (+)**	1.134 (0.675~2.254)	0.655	1.358 (0.728~2.513)	0.286
**Src-1**				
** Low**	Reference		Reference	
** High**	1.645 (1.023~2.679)	**0.048**	1.574 (1.013~2.286)	0.065
**Twist1**				
** Low**	Reference		Reference	
** High**	1.721 (1.179~3.334)	**0.021**	1.539 (1.095~3.084)	**0.017**
**Lymph node metastasis**				
** NO**	Reference		Reference	
** Yes**	1.011 (0.089~1.532)	0.435	1.232 (0.579~2.084)	0.501
**Distant metastasis**				
** NO**	Reference		Reference	
** Yes**	1.754 (1.106~2.913)	**0.012**	1.622 (1.064~2.563)	**0.014**

OS: Overall Survival; PFS, Progression-free Survival.

### Knockdown of Src-1 and Twist1 suppresses colony formation, anchorage-independent growth, cell migration, invasion and tumor xenografts growth of CNE-1 cells

**Fig 3:**
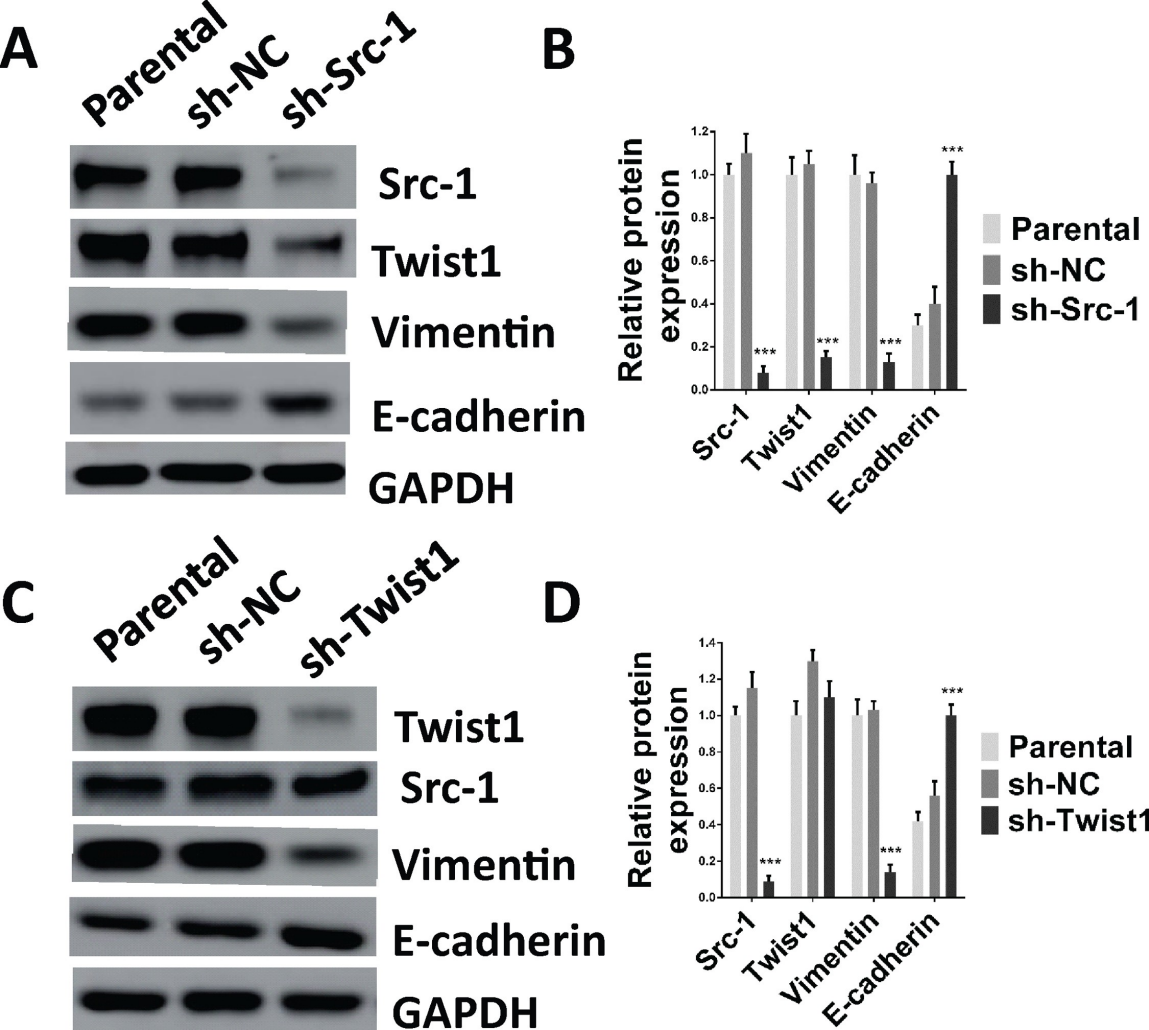
Knockdown of Src-1 or Twist1 expression in CNE-1 upregulates E-cadherin expression and downregulates Vimentin expression. **(A, B)** 2.5×10^5^ CNE-1 cells were seeded in 6-well-plates and transduced with sh-Src-1, sh-Twist1 or a negative control (sh-NC) for 4 days, then cell lysates were analyzed by western blot. **(C, D)** quantification of western blot bands in A and B. Protein expression was normalized to GAPDH. Lysates of parental CNE-1 cells were used as positive control. ***P<0.05 comparing with sh-NC group.

**Fig 4:**
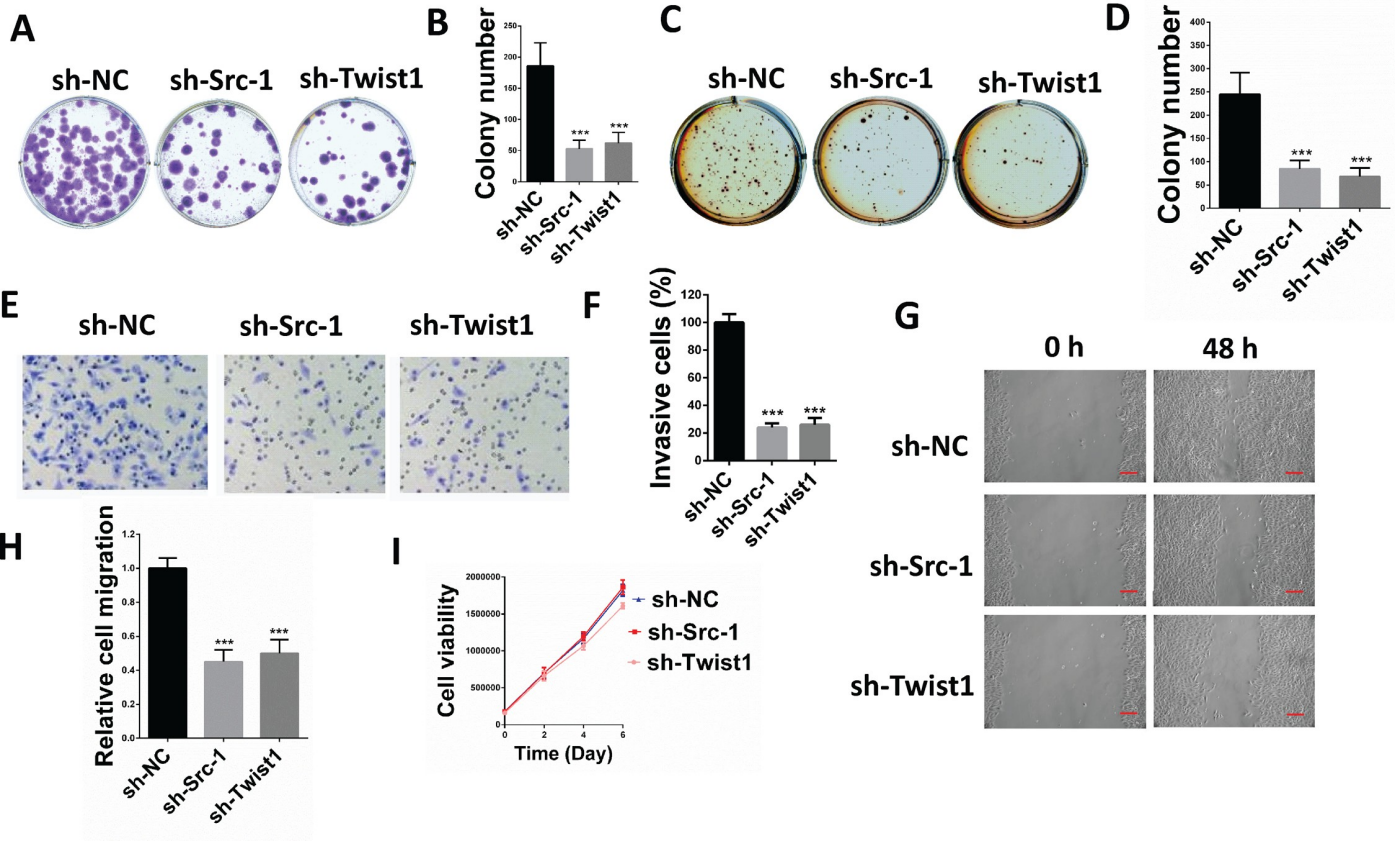
Knockdown of Src-1 or Twist1 expression in CNE-1 suppressed colony formation, anchorage-independent growth, cell migration and invasion. **(A)** 500 CNE-1 cells transduced with sh-Src-1, sh-Twist1 or sh-NC control were seeded in 6-well-plates, allowed grow for 2 weeks and stained with crystal violet. **(B)** Colony numbers from A. **(C)** 5000 CNE-1 cells transduced with sh-Src-1, sh-Twist1 or sh-NC control were seeded in soft agar and cultured for 3 weeks, then stained with 1 mg/ml MTT. **(D)** colony numbers from C. **(E)** 1×10^5^ CNE-1 cells transduced with sh-Src-1, sh-Twist1 or sh-NC control were used for transwell cell invasion assay, then stained with Giemsa dye and imaged after incubating for 24hr. **(F)** percentage of invasion cells from E comparing with sh-NC. **(G)** CNE-1 cells transduced with sh-Src-1, sh-Twist1 or sh-NC control were used for scratch wound healing assay, photos of the plates were took at 0h and 48h. Scale bars = 50um.**(H)** Relative cell migration from G analyzed by Digimizer software system. (**I**) cell proliferation of sh-Src-1, sh-Twist1 or sh-NC transduced CNE-1 cells was evaluated by cell viability assay. ***P<0.05 comparing with sh-NC group.

**Fig 5:**
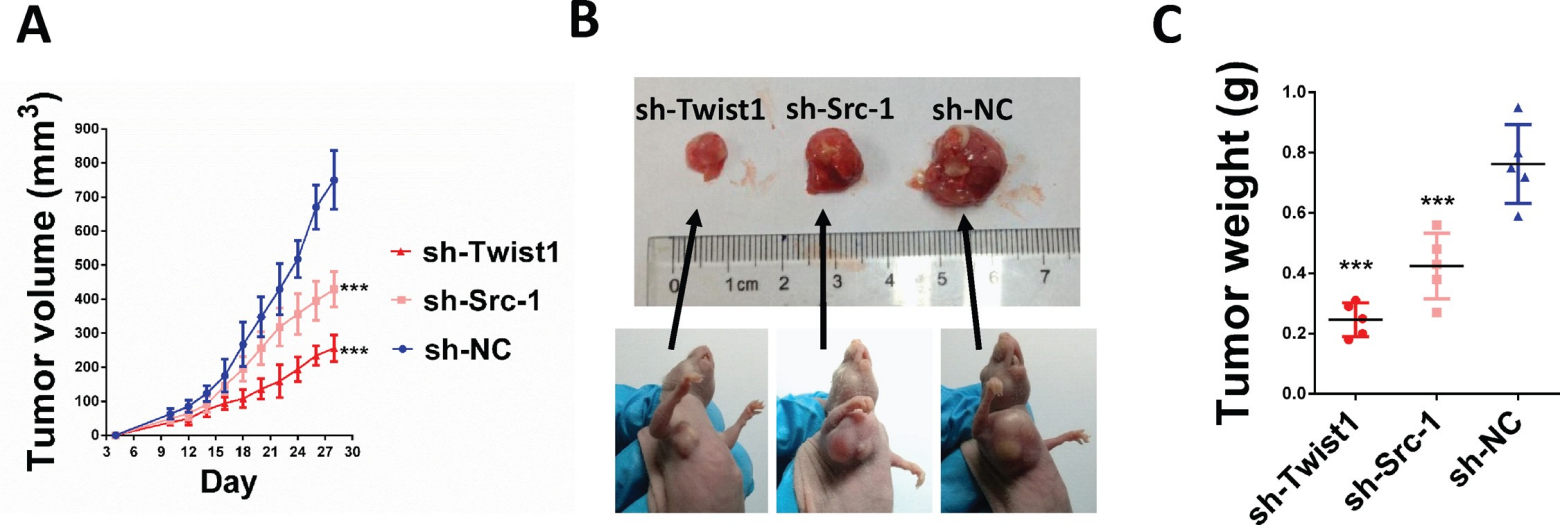
Knockdown of Src-1 or Twist1 expression suppressed tumor xenografts growth in CNE-1. 2×10^6^ CNE-1 cells expressing either sh-Src-1, sh-Twist1 or sh-NC cotrol were subcutaneously injected in the armpits of nude mice (n = 5). Tumor size were measured every two days (A), and four weeks after implantation, tumor xenografts were dissected out (B) and weighed (C). ***P<0.05 comparing with sh-NC group.

To determine the role of Src-1 and Twist1 in NPC development, a lentivirus-based shRNA expression system was used to suppress Src-1 or Twist1 expression in NPC cell line CNE-1. The protein expression of Src-1 or Twist1 in CNE-1 was significantly down-regulated by sh-Src-1 or sh-Twist1 (Fig 3A–3D). Moreover, downregulation of Src-1 repressed Twist1 protein expression, but downregulation of Twist1 had no influence on Src-1. This further demonstrated that Src-1 could regulate Twist1 expression. In addition, knockdown of Src-1 or Twist1 increased E-cadherin expression and inhibited expression of Vimentin (Fig 3A–3D). To study the influence of Src-1 and Twist1 knockdown in CNE-1, colony formation assay, soft agar assay, cell migration assay, cell invasion assay and tumor xenograft growth assay were conducted. Colony formation assay and soft agar assay revealed that knockdown of Src-1 or Twist1 markedly repressed colony formation (Fig 4A and 4B) and anchorage-independent growth of CNE-1 (Fig 4C and 4D). Furthermore, the invasion capacity of CNE-1 was largely impaired by knocking-down of Src-1 or Twist1 (Fig 4E and 4F). Knockdown of Src-1 or Twist1 also resulted in slower healing of scratch wounds inflicted on CNE-1 cells (Fig 4G and 4H). In addition, cell growth of sh-Src-1 or sh-Twist1 transduced CNE-1 cells were evaluated by cell viability assay, and found that there were no obvious difference in cell proliferation comparing with sh-NC transduced CEN-1 cells (Fig 4I). Besides, tumor xenografts growth of sh-Src-1 or sh-Twist1 transduced CNE-1 cells was significantly suppressed comparing with sh-NC transduced CNE-1 cells (Fig 5A). Tumor volume and weight of CNE-1 xenografts at four weeks after implantation were dramatically inhibited by knocking-down of Src-1 or Twist1 (Fig 5B and 5C).

### Enforced expression of Src-1 and Twist1 promotes anchorage-independent growth, migration, invasion and lung metastasis of HNE-2 cells

**Fig 6:**
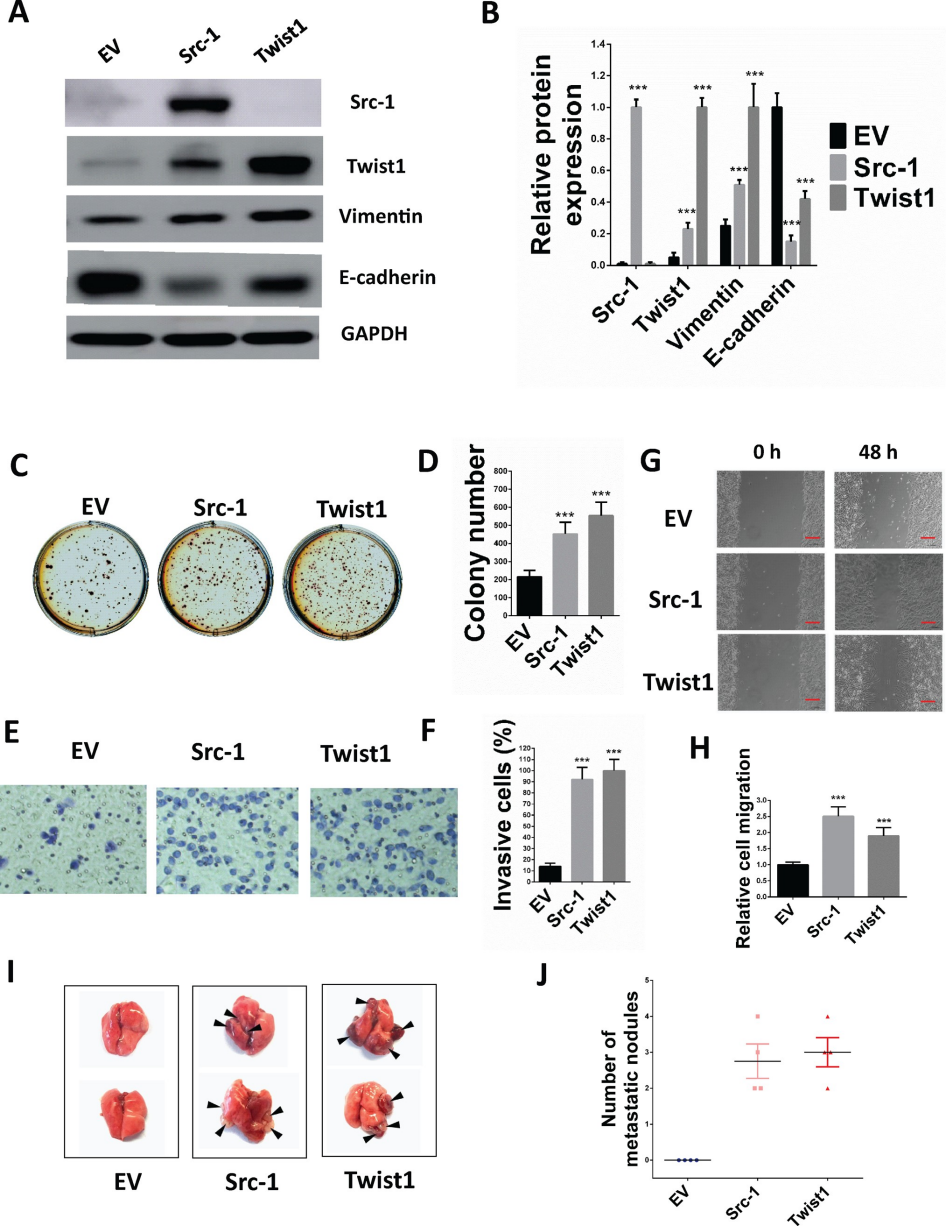
Enforced Src-1 or Twist1 expression in HNE-2 promotes anchorage-independent growth, migration, invasion and lung metastasis. **(A)** 2.5×10^5^ HNE-2 cells were seeded in 6-well-plates and transduced with Src-1 or Twist1 expression lentivirus for 4 days, then cell lysates were analyzed by western blot. Cells transduced with empty vector (EV) were used as control. **(B)** Quantification of western blot bands in A. Protein expression was normalized to GAPDH. **(C)** 5000 HNE-2 cells transduced with Src-1, Twist1 or EV control were seeded in soft agar and cultured for 3 weeks, than stained with 1 mg/ml MTT. **(D)** colony numbers from C. **(E)** 1×10^5^ CNE-1 cells transduced with Src-1, Twist1 or EV control were used for transwell cell invasion assay, then stained with Giemsa dye and imaged after incubating for 24hr. **(F)** percentage of invasion cells from E comparing with EV control. **(G)** HNE-2 cells transduced with Src-1, Twist1 or EV control were used for scratch wound healing assay, photos of the plates were took at 0h and 48h. Scale bars = 50um **(H)** Relative cell migration from G analyzed by Digimizer software system. (**I-J**) 3×10^6^ HNE-2 cells transduced with Src-1, Twist1 or EV control were injected into the tail vein of nude mice. Represent images of tumors metastasized to lung were shown (**I**). Number of tumors in lung was calculated (**J**). ***P<0.05 comparing with EV group.

To figure out whether Src-1 and Twist1 was involved in the progression of NPC, Src-1 and Twist1 expression plasmids were constructed and transduced into NPC cell line HNE-2. As shown in Fig 6A, Src-1 and Twist1 were successfully overexpressed in HNE-2, and enforced Src-1 expression also lead to upregulation of Twist1. Moreover, enforced Src-1 or Twist1 expression resulted in upregulation of Vimentin and downregulation of E-cadherin, suggesting that Src-1 and Twist1 induced EMT in HNE-2 (Fig 6A and 6B). To study the role of Src-1 and Twist1 overexpression in HNE-2, soft agar assay, cell migration and invasion assay *in vitro* and lung metastasis *in vivo* were conducted. Soft agar assay revealed that anchorage-independent growth of HNE-2 was increased by enforced expression of Src-1 and Twist1 (Fig 6C and 6D). Transwell invasion assay was conducted to evaluate the invasion ability and found that overexpression of Src-1 and Twist1 increased invasion of cells (Fig 6E and 6F). Moreover, scratch wound healing assay showed that enforced Src-1 and Twist1 expression enhanced migration capacity of HNE-2 (Fig 6G and 6H). To evaluate the lung metastasis ability of Src-1 or Twist1 transduced HNE-2, these cells were injected into the tail vein of nude mice and examined the tumors formed in lung. As expected, Src-1 and Twist1 dramatically increased the number of tumors formed in lung (Fig 6I and 6J). Therefore, these results demonstrated that enforced expression of Src-1 and Twist1 evoked EMT phenotype and consequently promoted anchorage-independent growth, migration, invasion *in vitro* and lung metastasis *in vivo* of HNE-2 cells.

## Discussion

In the present study, we have found that Src-1 and Twist1 were aberrantly upregulated in human NPC tissues, and upregulation of Src-1 or Twist1 was associated with advanced tumor stage, distant metastasis and unfavorable prognosis. Moreover, we found that knockdown of Src-1 or Twist1 in human NPC cell line CNE-1 suppressed colony formation, anchorage-independent growth, cell migration, invasion and tumor xenografts growth, while enforced expression of Src-1 or Twist1 in human NPC cell line HNE-2 promotes anchorage-independent growth, cell migration and invasion. In addition, Src-1 and Twist1 could suppress E-cadherin expression and increase Vimentin expression, thus suggested that Src-1 and Twist1 enhanced the malignant behaviors of NPC cells via inducing EMT. Our data indicated that Src-1 and Twist1 could be important biomarkers to predict outcome and potential therapy targets for NPC patients.

As a coactivator, the ability of Src-1 to coordinate multiple signaling pathways makes it plays an important role in tumor growth, metastasis and drug resistance. Src-1 expression is significantly upregulated in nearly 20% breast cancer, and is associated with positive HER2 expression, large and high grade tumor, disease recurrence, poor survival and resistance to endocrine therapy [15, 16]. In androgen-dependent prostate cancer, increased Src-1 expression is correlated with lymph node metastasis, and depletion of Src-1 in androgen-dependent prostate cancer cell line LNCaP represses AR-dependent cellular proliferation and activation of AR target genes [17]. In non-anaplastic thyroid cancer, high Src-1 expression is associated with poor cellular differentiation, capsular invasion and disease progression [18]. Serum levels of S100β are associated with tumor burden, poor prognosis and poor response to treatment in melanoma patients [29]. In cutaneous melanomas, Src-1 increases S100β expression by serving as a coactivator for HOXC11, and high expression of Src-1 and HOXC11 was found in malignant melanoma but not in benign nevi [30]. In our study, we have found that Src-1 was aberrantly upregluated in NPC patients, and high Src-1 expression was associated with higher tumor stage, distant metastasis and poor prognosis. High Src-1 expression was an independent predictive factors for unfavorable OS time of NPC patients. By inhibiting Src-1 expression in CNE-1 or overexpression Src-1 in HNE-2, we have found that Src-1 expression was associated with anchorage-independent growth, cell migration and invasion of NPC cells. These results indicated that high Src-1 expression could predict an unfavorable prognosis for NPC patients and promote migration and invasion of NPC cells *in vitro*.

Twist1 is aberrantly expressed in many types of aggressive tumors, including breast cancer [21], hepatocellular carcinoma [22], prostate cancer [23], gastric cancer [24], bladder cancer [31], pancreatic cancer [32], oesophageal squamous cell carcinoma [33] and gliomas [34]. Twist1 is significantly upregulated in bladder cancer tissues comparing with normal bladder tissues, and among the cancer tissues, Twist1 was remarkably higher in metastatic lesions comparing with primary tumors [31]. IHC staining and RT-PCR analysis revealed that Twist1 expression was increased in oesophageal squamous cell carcinoma, and high Twist1 expression was associated with greater metastasis risk and low E-cadherin expression [33]. In human gliomas, increased Twist1 expression is associated with high tumor stage, and overexpressed Twist1 in a human glioma cell line significantly enhanced cell invasion [34]. In addition, previous study has shown that Twist1 expression is directly associated with LMP1 expression and tumor metastasis clinically in NPC patients [35]. Corresponded with this, we have found that Twist1 was significantly upregulated in NPC patients in our study and high twist1 expression was associated with higher tumor stage, recurrence and distant metastasis. Besides, multivariate analysis revealed that high Twist1 expression was independent predictive factors for the unfavorable OS and PFS time. In the functional study, we have found that knockdown Twist1 expression significantly suppressed colony formation, anchorage-independent growth, cell migration, invasion and tumor xenografts growth of CNE-1 cells, and enforced Twist1 expression promoted anchorage-independent growth, cell migration and invasion of HNE-2 cells. Overall, these results demonstrated that aberrantly upregulated Twist1 expression was associated with poor prognosis, tumor migration and invasion, thus Twist1 could be prognostic biomarkers and potential therapy targets for NPC patients.

In our study, we have found a connection between Src-1 and Twist1 expression in NPC. Increased Src-1/Twist1 expression was found in 32.8% (44/134) NPC patients, and spearman analysis demonstrated a correlation between Src-1 and Twist1 expression. High Src-1 expression and high Twist1 expression were both associated with advanced tumor stage, distant metastasis and unfavorable prognosis. In the functional study, either high expression of Src-1 or Twist1 could promote anchorage-independent growth, cell migration and invasion of NPC cells. As previous reported, Src-1 could potentiate PEA3-mediated Twist1 expression in breast cancer [26]. Therefore, it was not surprising that repressed Src-1 expression in CNE-1 resulted in downregulation of Twist1, and enforced Src-1 expression in HNE-2 increased Twist1 expression in the western blot analysis. These results suggested that Src-1 regulated Twist1 expression in NPC cell lines, and elevated Twist1 expression in NPC patients might partially due to the increased expression of Src-1.

Almost 80% of life-threatening human malignancies derive from epithelial tissues, including nasopharyngeal carcinoma. Neoplastic cells from early tumors retain the key biological phenotypes of epithelial cells, such as lack of motility and an ability to form continuous cell sheets. In contrast, tumor cells in high aggressive primary tumors display mesenchymal features, such as high motility and invasiveness. Thus, epithelial-mesenchymal transition is crucial for cancer progression. EMT is characterized by decreasing of E-cadherin expression and increasing of N-cadherin and Vimentin expression in tumor cells. As previous reports, Twist1 could induce N-cadherin expression through Cul2 circular RNA, and suppress E-cadherin expression by binding directly to the E-cadherin promoter [36, 37]. In breast cancer, Src-1 could upregulate Twist1 expression, thus further suppress E-cadherin expression indirectly [26]. In our study, we have found that both Src-1 and Twist1 could suppress E-cadherin expression and increase Vimentin expression in NPC cell lines. Thus we speculated that Src-1 and Twist1 promoted cell migration and invasion of NPC cells by inducing EMT.

In summary, our study revealed that the expression of Src-1 and Twist1 was aberrantly upregulated in NPC tissues and was associated with advanced tumor stage, distant metastasis and unfavorable prognosis. Src-1 and Twist1 could promote anchorage-independent growth, cell migration and invasion of NPC cells *in vitro*, and this was possibly due to inducing of EMT process. Our results indicated that Src-1 and Twist1 could be potential prognostic biomarkers and therapy targets in NPC patients.

## Ethical approval

All procedures performed in studies involving human participants were in accordance with the ethical standards of the institutional and/or national research committee and with the 1964 Helsinki declaration and its later amendments or comparable ethical standards. Informed consent was obtained from all individual participants included in the study. All procedures performed in studies involving animals were in accordance with the ethical standards of the ethics committee of the Shenzhen People's Hospital at which the studies were conducted.

## Abbreviations

Src-1: Steroid receptor coactivator 1
NPC: nasopharyngeal carcinoma
IHC: immunohistochemistry
OS: overall survival
PFS: progression-free survival
EMT: epithelial-mesenchymal transition
qRT-PCR: quantitative real-time polymerase chain reaction
shRNA: short hairpin RNA
MTT: thiazolyl blue tetrazolium bromide
NKC: non-keratinizing carcinoma
EBV: Epstein-Barr virus
DMEM: Dulbecco's Modified Eagle Medium
